# Clinical and Hemodynamic Outcomes with Enalapril Orodispersible Minitablets in Young Children with Heart Failure Due to Congenital Heart Disease

**DOI:** 10.3390/jcm13174976

**Published:** 2024-08-23

**Authors:** Maja Bijelic, Milan Djukic, Vladislav Vukomanovic, Vojislav Parezanovic, Milica Lazic, Andrija Pavlovic, Sasa Popovic, Miro Parezanovic, Igor Stefanovic, Stefan Djordjevic, Sanja Ninic, Sergej Prijic, Bojana Bozic Cvijan, Ida Jovanovic, Milica Bajcetic, LENA Study

**Affiliations:** 1 Department of Cardiology, University Children’s Hospital, 11129 Belgrade, Serbia; bijelicmaja11@yahoo.com (M.B.); milandjukic62@gmail.com (M.D.); vparezan@gmail.com (V.P.); andrijapavlovic88@gmail.com (A.P.); igorstefanovic@yahoo.com (I.S.); stf.djordjevic@gmail.com (S.D.); bojanabozic87@gmail.com (B.B.C.); idaj@rcub.bg.ac.rs (I.J.); 2 Department of Pediatrics, Faculty of Medicine, University of Belgrade, 11129 Belgrade, Serbia; vvukomanovicdr@gmail.com (V.V.); sergej2804@gmail.com (S.P.); 3 Department of Cardiology, Institute of Mother and Child Health “Dr Vukan Čupić”, 11070 Belgrade, Serbia; milicalazic272@gmail.com (M.L.); pop0408@hotmail.com (S.P.); mlparezanovic@yahoo.com (M.P.); sniz@mts.rs (S.N.); 4 Institute of Pharmacology, Clinical Pharmacology and Toxicology, Faculty of Medicine, University of Belgrade, 11129 Belgrade, Serbia

**Keywords:** pediatric cardiology, heart failure, congenital heart disease, ACEIs, enalapril, orodispersible minitablets

## Abstract

**Background**: The angiotensin-converting enzyme inhibitor (ACEI) enalapril is often administered to infants and young children with heart failure (HF) in various dosing regimens and formulations not adapted for their age. **Methods**: This prospective, two-center, open-label 8-week study evaluated an age-appropriate formulation of orodispersible minitablets (ODMTs) of enalapril (0.25 mg and 1 mg) in children aged 0 to 6 years with HF due to congenital heart disease. An age/weight-based dosing schedule was followed. Measures of echocardiographic parameters, blood pressure, heart rate, modified Ross score, and biochemistry were obtained over the 8-week period. The following two groups were assessed: ACEI-naïve and ACEI-pretreated patients. **Results**: In total, 53 children (age range of 0.05 to 4.8 years) were enrolled and 29 were ACEI-naïve. The average enalapril dose was 0.098 mg/kg (0.06–0.17 mg/kg) in the naïve group and 0.15 mg/kg (0.07–0.3 mg/kg) in pretreated patients. After 8 weeks, the modified Ross score and left ventricular diastolic dimension (LVD) z-score showed a significant decrease in both groups (*p* < 0.005). During 8 weeks follow-up, there were no difference in the z-scores for the systolic blood pressure (*p* = 0.071) or heart rate (*p* = 0.146). **Conclusions:** Pediatric patients treated with ODMTs of enalapril for 8 weeks had favorable improvements in LVD and HF symptoms.

## 1. Introduction

An orodispersible minitablet (ODMT) formulation of enalapril is in development for use in newborns, infants, and young children who cannot swallow solid forms designed for adults. Currently, enalapril is only available in tablet form at doses designed for the adult population, with no stable pharmaceutical forms tailored to the youngest age group. In everyday practice, enalapril tablets designed for adult patients are either divided or modified by crushing them and constituents are added to create powders or solutions. Such improvised preparation of drugs administered to children contributes to the risk of therapy inefficacy and unpredictable safety [1]. In addition, the lack of availability of commercial pediatric drug formulations licensed for children has a considerable influence on the complexity of medication regimens. Indeed, medication regimen complexity index scores were found to be higher in neonates, toddlers, and children than in adolescents with heart failure (HF) [2].

Despite the proven efficacy of the angiotensin-converting enzyme inhibitor (ACEI) in the treatment of adults with HF, evidence for their efficacy in treating HF in children is inconclusive and is based on only a few studies with mostly contentious designs and small sample sizes in the newborn and infant populations [3]. Moreover, there are no clear recommendations regarding the enalapril dosage in children, as confirmed by a European survey [4]. Due to the lack of adequate research, therapeutic strategies for treating HF in the pediatric population largely rely on extrapolating data obtained from studies conducted in the adult population [3,5]. Considering the differences in the pathophysiology of HF between children and adults, as well as the fact that maturation and developmental changes in children can influence pharmacokinetics and pharmacodynamics, extrapolating data from adults may lead to dosing errors [3,6]. The European Medicines Agency (EMA) and American Heart Association (AHA) do not recommend enalapril in neonates and children with HF due to the lack of efficacy and safety data [7,8]. However, enalapril is widely used for the treatment of HF in children in clinical practice, even with a body weight below 20 kg, and such patients currently receive enalapril off-label using age-inappropriate unlicensed formulations [7,9]. The pharmacotherapy of HF due to congenital heart disease plays a key role in reducing symptoms, stabilizing patients before surgery or heart transplantation, and potentially delaying the need for surgery [6]. Therefore, the development of ACE inhibitor formulations that are adequate for use in the youngest pediatric age group is essential. The European Commission approved funding for the LENA (Labeling of Enalapril from Neonates up to Adolescents) project in 2013 for the development and evaluation of an innovative ODMT formulation of enalapril for the treatment of HF [10]. Within the LENA project, ODMTs contain 0.25 mg and 1 mg of enalapril maleate, with a diameter of 2 mm. ODMTs are designed to quickly dissolve upon contact with water and saliva, and are more convenient for use in pediatric patients [10]. The results published so far from the LENA study have revealed notable differences in the pharmacokinetics of enalapril ODMTs among children with heart failure, particularly those with congenital heart disease. In the youngest age group, especially infants under 1 year old, the time to reach the maximum plasma concentration (Tmax) is significantly longer, at 2 h, compared to 1 h in older children. This extended Tmax in infants may be due to developmental changes in the gastrointestinal tract, affecting how the medication is absorbed and processed. These results emphasize the need to account for age-related physiological differences when administering medications to pediatric heart failure patients [11]. The aim of our study was to assess the effectiveness of this new formulation in dosing regimens for the youngest pediatric age groups (0 to 6 years).

## 2. Materials and Methods

### 2.1. Study Design

This prospective, phase II/III open-label study was conducted at the Department of Pediatric Cardiology of the University Children’s Hospital, Belgrade, and the Institute of Mother and Child Health “Dr Vukan Čupić”, Belgrade, Serbia, as a part of the LENA study. The study design was approved by the ethics committee at each institution (University Children’s Hospital in Belgrade, 29 February 2016, No. 26/307, and the Institute of Mother and Child Health “Dr Vukan Cupic”, 5 April 2017, No. 8/9). Additionally, informed consent from parents or legal guardians was obtained prior to enrolling any participants. The study was conducted in accordance with the principles outlined in the Good Clinical Practice Guideline and the Declaration of Helsinki and its subsequent amendments.

### 2.2. Inclusion/Exclusion Criteria

Children diagnosed with HF with left-to-right shunts due to congenital heart disease (CHD) were eligible for inclusion from birth to younger than 6 years with a body weight of at least 2.5 kg or more. Included patients could have either been ACEI-naïve or previously treated with ACEIs. Patients were ineligible for inclusion if they exhibited severe HF and/or end-stage HF that would hinder the initiation or continuation of ACEIs, had blood pressure levels too low for their age (less than the fifth percentile), suffered from restrictive or hypertrophic cardiomyopathies, had valvular obstruction (with a peak systolic gradient exceeding 30 mm Hg), had uncorrected severe peripheral stenosis of large arteries such as severe aortic coarctation, severe renal impairment characterized by a serum creatinine level exceeding twice the upper limit of normal, had a history of angioedema, or demonstrated hypersensitivity to ACEIs.

### 2.3. Study Drug Administration

The study’s investigational medicinal products were ODMTs that contained either 0.25 mg or 1 mg of enalapril maleate (Ethicare GmbH, Haltern am See, Germany). The ODMT was administered to the patient by inserting it into the buccal cavity. A drink of the patient’s/parent’s choice (e.g., breast milk, infant formula, cow’s milk, or water) was used to facilitate swallowing. In the case of very low doses (<0.25 mg), a protocol was devised to prepare enalapril dispersions using a single 0.25 mg ODMT using an oral syringe.

Patients received ODMTs through individually tailored titration according to a specifically defined dosing regimen. Patients received an initial dose of enalapril in the morning of day 1 and blood pressure was monitored for 8 h to detect any significant decrease. If the dose was well tolerated, it was maintained as the initial dose for the following 1 to 6 days. In children older than 3 years of age, a second evening dose was also given for greater therapeutic effect. The initial dose was gradually increased (up-titrated) until reaching the planned target dose and/or the maximum tolerated dose. Once the optimal dose was achieved, patients continued to receive this dose throughout the 8-week study period, with visits scheduled at least once or twice per week.

Patients who were previously treated with ACEIs were transitioned to an equivalent dose of enalapril, and from there, the dose could potentially be up-titrated to the maximum tolerated dose based on the investigator’s clinical judgment and prescription. The ODMT dose in ACEI-naïve patients was adjusted upwards based on the investigator’s discretion and prescription, following the protocol. For children from 1 day old to under 6 months old (weighing between 2.5 and 7 kg), the recommended initial and first dose was 0.25 mg of enalapril once daily. However, very young and low-weight patients, for whom the initial dose of 0.25 mg enalapril was deemed excessively high by the investigator, received a reduced initial dose, such as 0.025 mg of enalapril (10% of 1 × 0.25 mg, equivalent to 0.01 mg/kg/day administered once daily), or 0.125 mg of enalapril (50% of 1 × 0.25 mg), up to a maximum of 4 × 0.25 mg (0.4 mg/kg/day administered twice daily). For children from 6 months to under 3 years old (weighing between 8 and 15 kg), the recommended initial and first dose was 1 × 0.25 mg of enalapril once daily. For children aged 3 to less than 6 years (weighing between 16 and 25 kg), the recommended initial dose was 1 × 0.25 mg of enalapril, and the first dose was 2 × 0.25 mg of enalapril two times a day. The proposed dosing regimen for this pediatric clinical study was determined using modeling and simulation, with the goal of achieving a similar exposure in the pediatric age subgroups as observed in adults [11].

### 2.4. Assessments

The design of the study included the following visits: screening visit (lasting up to 21 days), first drug administration visit (which could be combined with the screening visit for patients who were not previously on ACEI therapy or whose initial dose was 50% higher than the 0.25 mg ODMT), titration visit(s) (as needed—up to a maximum of four), dose-confirmation visit (which could be combined with the first follow-up visit), three follow-up visits (every two weeks), and a final visit (day 56 or the last visit as part of the early study termination).

At the screening visit and visits during the ODMT administration, data were collected on the medical history, clinical examination, body weight and stature, essential physiological functions (heart rate and blood pressure), sampling of N-terminal pro-brain natriuretic peptide (NT-proBNP), plasma creatinine, serum potassium, and ECG. Weight-for-age and height-for-age z-scores were calculated using the World Health Organization standards [12]. To assess the severity of HF, a modified Ross score classification [13] was utilized. Each patient’s modified Ross score was assessed at baseline and at the visits during the ODMT administration by a pediatric cardiologist. Echocardiographic examination was conducted during the screening visit and final visit. The measurements of three consecutive cardiac cycles were averaged for analysis. Standard measurements, with a recording of findings, were conducted during each echocardiographic examination. The following techniques were used for measurements: 2D, M-mode, and Doppler techniques. Standard echocardiographic measurements included systolic and diastolic dimensions of the left ventricle and fractional shortening (PSLAX), transvalvular flow velocity through the aorta (AP4Ch), pulmonary flow velocity (PSSAX), flow velocity through the mitral valve (E), velocity in the jet of mitral regurgitation (AP4Ch), flow velocity through the tricuspid valve (E), and velocity in the jet of tricuspid regurgitation (AP4Ch). Echocardiographic measurements and derived indices were expressed as z-scores relative to the body surface area or age in healthy children [14].

Blood pressure and heart rate were measured in a seated or lying position after 5 min of rest. The same instrument (calibrated) was used for blood pressure measurements. Measurements were always conducted on the same arm, using an appropriately sized cuff. Blood pressure was measured every 30 min over an 8 h period during the first drug administration visit, over 4 h during the first titration visit, and over 2 h during subsequent titration visits. Dose titration or therapy administration were not conducted in children aged up to 6 years with a blood pressure of less than 70 mmHg + 2 × years of age. Systolic blood pressure (SBP) and diastolic blood pressure (DBP) were expressed as SBP z-scores (SBP-Z) and DBP z-scores (DBP-Z), adjusted for the age, gender, and height of each individual [15].

### 2.5. Statistical Analysis

Continuous data are presented as mean ± SD or as median with min–max, depending on the normality of distribution. Variables were assessed for normality using the Kolmogorov–Smirnov test. Categorical data are expressed as numbers with percentages. Comparisons between pre-enalapril and follow-up measurements were conducted using either a paired sample *t*-test or a Wilcoxon signed-rank test for non-normally distributed values. A *p*-value < 0.05 was considered significant. One-way ANOVA was used for comparisons between multiple groups with a post hoc analysis with a Bonferroni test. Mann–Whitney and Kruskal–Wallis tests were used for non-parametric data. A mixed-way ANOVA was used to show the influence of time and treatment and a Friedman 2-way ANOVA test was used for non-normally distributed values. Statistical analysis was performed using SPSS software version 23 (IBM Corp, released 2015; IBM SPSS Statistics for Windows, Version 23.0; Armonk, NY, USA: IBM Corp).

## 3. Results

### 3.1. Patient Characteristics

A total of 53 patients who had a diagnosis of HF secondary to CHD were screened and enrolled. In total, seven patients had Down syndrome. Patient characteristics at baseline are shown in Table 1. Children up to 12 months of age represented 92.4% of all treated patients. In total, 29 patients were ACEI-naïve; all were younger than 12 months. The remaining patients (24) had previously received an ACEI: 18 received prior captopril and 6 received enalapril. Almost all of the patients (52) were receiving furosemide (1 mg/kg/day) and spironolactone (1 mg/kg/day) as concomitant medications, with 10 patients on milrinone for 1–2 days after cardiac surgery, 2 on digoxin, and 1 on dopamine.

Twenty-three patients had both ventricular and atrial septal defects, while fourteen had isolated ventricular septal defects (VSD), seven had atrioventricular septal defects (AVSD), three had patent ductus arteriosus (PDA), three had dextro-transposition of the great arteries (D-TGA) with a VSD, two had double-outlet right ventricle (DORV) with a VSD, and one had common arterial trunk. Fifteen patients underwent cardiac surgery during the study period. Ten patients underwent surgical correction for VSD, at an average age of 5 months (range of 3 to 8 months). Surgery was also performed on one patient with PDA, one patient with D-TGA plus VSD, two patients with DORV plus VSD, and one patient with common arterial trunk. Despite the surgical correction of CHD, most of the operated patients continued the enalapril ODMT therapy. Two operated patients continued ODMTs until the end of the study due to mitral regurgitation after surgery. Eight patients continued the ODMTs due to residual VSD. Three patients were operated on only 2–3 days before the end of the study. After surgery, two patients discontinued the therapy after 28 and 42 days of the enalapril ODMT treatment. Premature drug discontinuations occurred in one patient as a result of hypotension and in one patient due to worsening HF.

### 3.2. Dosing

**Figure 1:**
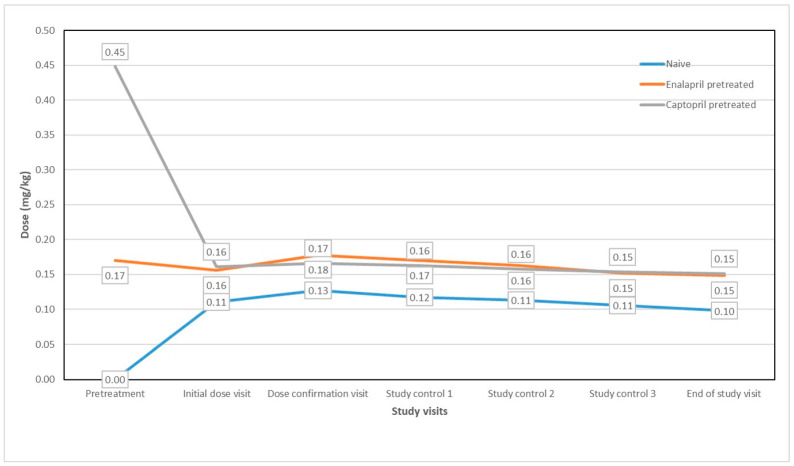
Average doses of the ODMT of enalapril received over the study period.

Most ACEI-pretreated children were transitioned to equivalent doses of enalapril ODMTs, with a minority to different dose levels. The average dose of captopril administered to pretreated patients was 0.448 ± 0.16 mg/kg, which corresponds to an enalapril dose of 0.18 mg/kg (Figure 1). This average dose of captopril in pretreated patients was 1.6 times higher than the average initial dose ODMT of enalapril in the naïve group (0.11 mg/kg). Average doses of enalapril in pretreated patients were not statistically significantly higher than the dose of the ODMT of enalapril received (*p* = 0.089). The average dose of the ODMT of enalapril was 0.098 mg/kg (0.06–0.17 mg/kg) in the naïve group and 0.15 mg/kg (0.07–0.3 mg/kg) in the pretreated group. The pretreatment group consistently exhibited a statistically significant higher doses of the ODMTs compared to the naïve patient group throughout the observation period (*p* < 0.001). Of note, 33% of participants who were previously on a three-times-daily pretreatment regimen switched to using ODMTs twice a day.

According to the LENA study protocol, the principal investigator had the discretion to titrate the dose up to the maximum tolerated level while continuously monitoring safety parameters such as the blood pressure, serum creatinine, and potassium levels. The principal investigator decided to titrate the medication dose for seven patients due to the clinical findings observed during the initiation of the enalapril therapy. The characteristics of the titrated patients are provided in Table 2.

### 3.3. Effects of the ODMT of Enalapril on Clinical, Echocardiographic, and Hemodynamic Parameters

After 8 weeks of treatment with the enalapril ODMTs, the modified Ross score showed a significant decrease overall (*p* < 0.001), and in both the ACEI-pretreated (*p* < 0.005) and ACEI-naïve patients (*p* < 0.001) (Table 3). When the patients who underwent surgery were excluded, a statistically significant decrease in the modified Ross score value was seen from the screening visit to the end-of-study visit among the non-operated patients (4.05 ± 3.17 vs. 2.15 ± 2.19, *p* < 0.05). In children under 1 years of age, there was also a statistically significant decrease in the modified Ross score value from the screening visit to the end-of-study visit (4.69 ± 2.81 vs. 2.18 ± 2.05, *p* = 0.001).

Weight-for-age z-scores analyzed at the screening and final study visits did not show a significant difference. However, there was a statistically significant difference in the height-for-age z-scores (*p* = 0.008).

The left ventricular diastolic dimension (LVD) z-score also decreased significantly after the treatment with the enalapril ODMTs overall (*p* < 0.001), and in the pretreated group (*p* = 0.004) and the naïve group (*p* = 0.01) (Table 3). When patients who underwent surgical intervention were excluded, there was a statistically significant decrease in the LVD z-score among non-operated patients from the screening visit to the end-of-study visit (1.82 ± 1.73 vs. 1.31 ± 1.48, *p* < 0.05). When observing the difference between ACEI-naïve and ACEI-pretreated, non-operated patients, a statistically significant reduction in the diastolic dimension of the left ventricle was found in the naïve-patient group (*p* = 0.008).

Further details on the effects of the enalapril ODMTs on the echocardiographic measurements are given in Table S1.

**Figure 2:**
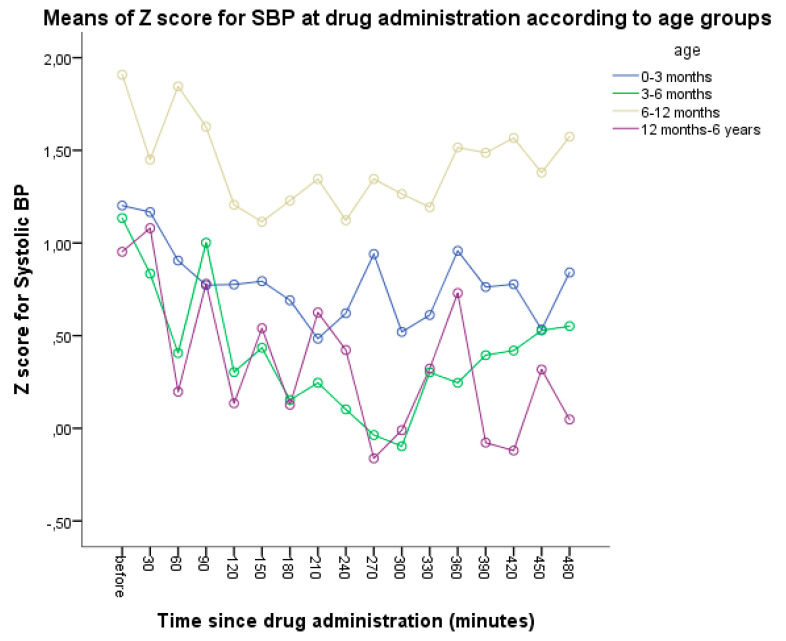
The z-score for systolic blood pressure during the initial phase.

During the initial phase, the SBP values, as well as the z-score of the SBP, were the lowest at the 300th minute after the ODMT administration in both the pretreated and naïve groups. The SBP z-score changes during the initial phase are presented in Figure 2. The z-score of the SBP was the lowest in the youngest age group (0–3 months) at the 210th minute, in the 3–6 months age group at the 300th minute, in the 6–12 months age group at the 240th minute, and in the oldest age group (12 months to 6 years) at the 270th minute. Across the five patient monitoring visits and measurements of hemodynamic parameters, the z-scores of the SBP did not exhibit statistically significant differences over time (*p* = 0.071) (Table 4). Further details on the effects of the enalapril ODMTs on the SBP and DBP, and by pretreatment group and age, are shown in Figures S1 and S2.

During an 8 h monitoring of the heart rate, there was no statistically significant difference in the heart rate values over time both in naïve patients (*p* = 0.426) and pretreated patients (*p* = 0.227) (Figure S3). In naïve patients, the lowest heart rate value was recorded at the 210th minute after the ODMT administration, while, in pretreated patients, it occurred at the 180th and 300th minutes. During the monitoring across five visits, despite showing a declining trend, there was no statistically significant difference in the heart rate values (*p* = 0.146) (Table 4). Further details on the effects of the enalapril ODMTs on the heart rate by the pretreatment group and age are shown in Figure S4.

Analyzing the differences in the hemodynamic parameters at the end of the study between the group of naïve, non-operated patients and the pretreated, non-operated patients, no statistically significant differences were observed in the z-score values for the systolic blood pressure (*p* = 0.810) or heart rate values (*p* = 0.134).

The NT-proBNP values showed statistically lower values after 8 weeks of the enalapril ODMT treatment in all of the participants: the median NT-proBNP value was 1111 pmol/L at the screening visit and 484 pmol/L at the end-of-study visit (Table 5). Moreover, the decreasing trend in the median NT-proBNP values were observed not only in the ACEI-pretreated but also in the ACEI-naïve non-operated patients.

## 4. Discussion

This is the first study to assess the clinical effects of an innovative ODMT formulation of enalapril in children from birth up to 6 years old, with the vast majority aged under 12 months. Due to the availability of two low-dose ODMTs, precise doses could be tailored to the young patients’ age and weight. It was observed that lower equivalent doses of the ODMT of enalapril could be used in patients previously prescribed captopril. With current administration of extemporaneous oral preparations, the actual ACEI dose received is often uncertain, as preparations tend to have unknown bioavailability and stability compared to the original tablet. Importantly, one-third of participants who were previously on a three-times-daily pretreatment regimen switched to using ODMT twice daily in the present study. This may help improve convenience for the patient, parents, and for healthcare professionals if the child is in-hospital.

Positive clinical effects were observed after the administration of the ODMT of enalapril for 8 weeks, with a decrease in the modified Ross score values in the naïve and pretreated groups. Decreases in the modified Ross scores were accompanied by a decrease in the NT-proBNP values, which may be useful for predicting outcomes [16]. In addition, the enalapril ODMT treatment led to significant improvements in the echocardiographic findings, showing a reduction in the diastolic dimension of the left ventricle in the naïve and pretreated groups. During the initial phase of drug administration, the expected decrease in blood pressure occurred, but there was no significant hypotension during the initial or follow-up phases. Furthermore, there were no significant changes in heart rate throughout the entire 8-week monitoring period.

Other small studies have investigated enalapril in children, across a wide range of doses, with results that are consistent with the LENA study [17,18,19]. Sluysman et al. demonstrated positive therapeutic effects following the administration of a single dose of intravenous enalaprilat of 0.02 mg/kg followed by once-daily oral enalapril of 0.16 mg/kg/day for 7 days to eight infants aged up to 10 months diagnosed with HF due to a large VSD with left-to-right shunt [17]. A 27% decrease in left-to-right shunt was observed, and infants exhibited improved feeding and weight gain after medication administration, with no significant changes in heart rate. In a study of 35 infants and children with congestive HF associated either with residual mitral or aortic regurgitation following intracardiac repair (24 patients) or with dilated cardiomyopathy (11 patients), enalapril, at an average dose of 0.24 mg/kg, resulted in a decrease in LVD, with improved fractional shortening and systolic time intervals [19]. Positive echocardiographic effects have also been seen with captopril [20,21,22]. In a study of 12 children with aortic regurgitation or mitral regurgitation (aged 0.3 to 16 years) who received similar doses as in the LENA study (0.15–0.4 mg/kg/day; max. 5 mg/day), but over a longer period (from 1.1 to 8.8 years), there was a reduction in the volume overload of the left ventricle as well as myocardial thickness [21].

Not all studies with ACEIs in children have had wholly positive results. In a study of 63 patients with HF (median age was 5.4 months) who received enalapril of 0.30 mg/kg/day, 39 patients (58%) improved based on clinical, radiological, and laboratory data, 20 (30%) showed no improvement, and 8 (12%) had adverse effects requiring discontinuation of enalapril, with a significant decrease in blood pressure, which may reflect the non-individualized enalapril dose used [23]. Therapeutic benefit was not observed with enalapril of 0.2 to 0.3 mg/kg/day in a randomized, double-blind, placebo-controlled crossover trial involving 18 asymptomatic patients diagnosed with univentricular heart pathology who had previously undergone the Fontan procedure [24]; however, the hemodynamic characteristics of patients with single-ventricle physiology differed from those of the current population. Another study involving children with univentricular heart pathology also failed to demonstrate positive effects with enalapril of 0.4 mg/kg/day [25]. In an open-label, dose-finding study, 10 infants diagnosed with congestive HF were administered enalapril orally as an extemporaneously prepared suspension at a maximum dose of 0.08 mg/kg/day once daily [26]. Positive effects were not observed, which may be due to the low dose, low bioavailability, and the short duration of action of the preparation, as adequate drug blood concentrations were not achieved or maintained [26]. The positive outcomes seen in the LENA study indicate the need for a specific dosing regimen of a stable, easily titrated, age-appropriate enalapril formulation with the appropriate selection of the patient population.

In the LENA study, the majority of patients who underwent surgery had a diagnosis of VSD. The average age at the time of surgical intervention for these patients was 5 months, which is in line with the recommendations from the literature for VSD surgery in young infants with HF [27]. Other patients with the same diagnosis underwent surgery either immediately after the end of the study or later. This supports the fact that a good response to medical therapy contributes to hemodynamic stabilization, symptom regression, and, eventually, by delaying surgical intervention, may contribute to better procedural outcomes in the long-term follow-up.

Our study has several limitations. In this relatively small population, patients had CHD of varying complexity, which might affect the severity of HF. In addition, the modified Ross score might have been affected by parents’ impression of the child’s symptoms. Lastly, since several cardiologists performed echocardiographic examinations, there might be interobserver differences in the echocardiographic findings.

## 5. Conclusions

Enalapril ODMTs, administered using a specific dosing regimen, showed significant improvements in symptom regression and LV dimensions without significant blood pressure and heart rate changes in young children with HF due to CHD.

## Figures and Tables

**Table 1:** Demographics of the pediatric population with heart failure due to congenital heart disease.

Patient Group	*N*	Gender (m)	Gender (f)	Age (years)	Body Weight (kg)	Height (cm)
Total	53	26 (49.10%)	27 (50.90%)	0.49 (0.05–4.80)	5.45 (2.51–21.00)	61.31 (48–101)
1 day to 3 months	21 (39.60%)	11 (42.30%)	10 (37.00%)	0.12 (0.05–0.22)	3.80 (2.51–5.26)	54.76 (48.00–61.00)
3 months to 6 months	19 (35.80%)	10 (38.50%)	9 (33.30%)	0.34 (0.26–0.50)	4.78 (3.29–7.20)	59.28 (51.50–68.50)
6 months to 12 months	9 (17%)	3 (11.50%)	6 (22.20%)	0.61 (0.50–0.70)	6.90 (5.48–8.70)	68.92 (64.00–78.00)
12 months to 6 years	4 (7.50%)	2 (7.70%)	2 (7.40%)	2.78 (1.03–4.80)	13.97 (7.30–21.00)	88.12 (67.50–101.00)
Prior enalapril	6 (11.30%)	4 (15.40%)	2 (7.40%)	0.276 (0.07–0.40)	4.75 (3.10–6.70)	60.83 (52.00–67.00)
Prior captopril	18 (34.00%)	8 (30.80%)	10 (37.00%)	0.91 (0.10–4.80)	7.11 (3.40–21.00)	67.66 (53.00–101.00)
Treatment-naïve	29 (54.70%)	14 (53.80%)	15 (55.60%)	0.26 (0.04–0.70)	4.56 (2.51–7.22)	57.45 (48.00–71.00)

Values are given as mean (range) or frequencies (percent).

**Table 2:** Titration scheme in seven patients.

Screening Visit					Initial Dose Visit	Titration Visit 1	Titration Visit 2
Diagnosis	Age (days)	Weight (kg)	Pretreatment Dose (mg/kg)	NT-ProBNP (pmol/L)	ODMT Dose (mg)	ODMT Dose (mg)	ODMT Dose (mg)
VSD	85	4.01	Enalapril 0.15	2322	1 × 0.25	2 × 0.25	–
VSD + ASD	213	5.48	Naïve	1090	2 × 0.25	2 × 0.5	–
VSD + ASD	55	4.05	Naïve	811	1 × 0.25	2 × 0.25	–
VSD + ASD; Down sy	132	4.07	Captopril 0.25	756	2 × 0.25	2 × 0.5	–
VSD + ASD	117	4.02	Captopril 0.22	611	2 × 0.25	2 × 0.5	–
AVSD; Down sy	251	7	Naïve	1111	2 × 0.25	2 × 0.5	2 × 1
VSD + ASD	109	4.21	Naïve	2376	1 × 0.25	2 × 0.25	–

VSD = ventricular septal defect; ASD = atrial septal defect; AVSD = atrioventricular septal defect.

**Table 3:** Clinical and echocardiographic parameters.

	Screening Visit			End-of-Study Visit			*p*-Value
	Mean ± SD	Median	IQR	Mean ± SD	Median	IQR	
**Ross score**							
*All patients*	4.33 ± 2.94	5	2–7	2.02 ± 2.03	2	0–3	<0.001
*Pretreated*	3.08 ± 3.02	2.5	0–5.7	1.37 ± 1.73	0.50	0–3	<0.005
*Naïve*	5.45 ± 2.42	6	3.5–7	2.62 ± 2.11	2	1–4	<0.001
**Weight-for-age z-score**	−1.79 ± 1.81	−1.86	−2.80–(−0.76)	−1.75 ± 1.65	−2.00	−2.85–(−0.69)	0.841
**Height-for-age z-score**	−0.92 ± 1.87	−0.91	−1.66–(−0.34)	−0.24 ± 1.91	−0.48	−1.72–1.04	0.008
**LVD z-score**							
*All patients*	1.95 ± 1.68	2.05	1.14–3.20	1.27 ± 1.36	1.30	0.52–2.01	<0.001
*Pretreated*	2.2 ± 1.65	2.44	1.11–3.40	1.46 ± 1.33	1.47	0.78–2.42	0.004
*Naïve*	1.75 ± 1.70	1.79	1.11–2.81	1.11 ± 1.39	1.23	0.29–2.01	0.001
*Pretreated non-operated*	1.91 ± 1.73	1.86	0.28–3.05	1.50 ± 1.49	1.49	0.20–2.19	0.071
*Naïve non-operated*	1.72 ± 1.77	1.78	1.30–2.85	1.12 ± 1.50	1.50	0.58–2.13	0.008
**LVS z-score**	1.09 ± 1.55	1.40	0.16–2.23	0.74 ± 1.27	1.04	−0.55–1.5	0.038
**Shortening fraction%**	41.45 ± 5.28	41	37–45	39.81 ± 4.71	40	36–44	0.062

LVD = left ventricular diastolic diameter; LVS = left ventricular systolic diameter. SD = standard deviation; IQR = interquartile range.

**Table 4:** Effects of orodispersible minitablets of enalapril on the hemodynamic parameters.

Parameter (Mean ± SD)	Screening Visit	Dose-Confirmation Visit	Study Control Visit 1	Study Control Visit 2	Study Control Visit 3	End-of-Study Visit	*p*-Value
SBP z-score	1.19 ± 1.25	0.81 ± 1.11	0.52 ± 1.07	0.72 ± 1.07	0.67 ± 1.21	0.84 ± 1.22	0.066
DBP z-score	1.18 ± 1.24	1.23 ± 1.11	0.91 ± 0.92	1.08 ± 0.96	1.06 ± 0.95	0.93 ± 0.82	0.242
Heart rate	140.59 ± 19.47	137.71 ± 17.65	139.27 ± 19.22	138.73 ± 20.26	135.29 ± 16.72	135.19 ± 18.31	0.146

**Table 5:** N-terminal pro-brain natriuretic peptide (NT-pro BNP, pmol/L) pre-dose at the initial dose visit and at the end-of-study visit in all patents, and in ACEI-naïve and pretreated non-operated patients.

	Screening Visit			End-of-Study Visit			
	Mean	Median	Min–Max	Mean	Median	Min–Max	*p*-Value
All patients	2549.8 ± 3099.3	1111	8–11,056	906.2 ± 1058.9	484	41–5348	<0.005
ACEI-naïve non-operated	3043.2 ± 2057.5	2057.5	8–10,511	931.9 ± 887.5	596	41–2630	0.001
ACEI-pretreated non-operated	2030.5 ± 2901.1	711	45–11,056	879.15 ± 1238.7	383	68–5348	0.002

## Data Availability

The data presented in this study are available upon reasonable request from the corresponding author.

## Supplementary Materials

The following supporting information can be downloaded at: https://www.mdpi.com/article/10.3390/jcm13174976/s1. Table S1. Effects of orodispersible minitablets of enalapril on echocardiographic parameters. Figure S1. Effects of orodispersible minitablets of enalapril on z score for systolic blood pressure (SBP). Figure S2. Effects of orodispersible minitablets of enalapril on z score for diastolic blood pressure (DBP). Figure S3. Effects of orodispersible minitablets of enalapril on heart rate (HR) in the initial phase in naïve and pretreated patients. Figure S4. Effects of orodispersible minitablets of enalapril on heart rate (HR).
