# Supplementary material for: Clinical and Hemodynamic Outcomes with Enalapril Orodispersible Minitablets in Young Children with Heart Failure Due to Congenital Heart Disease
Source: J Clin Med. 2024 Aug 23;13(17):4976. doi: 10.3390/jcm13174976 (PMC11396157; doi:10.3390/jcm13174976)

Supplement

Table S1. Effects of orodispersible minitablets of enalapril on echocardiographic parameters

**Parameter (Mean±SD)**	**Screening visit**	**End-of-study visit**	***p* value**
Flow velocity through the aorta (m/s)	1.19±0.30	1.28±0.39	0.123
Pulmonary flow velocity (m/s)	1.81±0.67	1.69±0.82	0.103
Flow velocity through the mitral valve (E wave; m/s)	1.27±0.04	1.13±0.26	0.010*
Velocity in the mitral regurgitation jet (m/s)	0.99±1.78	0.89±2.07	0.684
Flow velocity through the tricuspid valve (E wave; m/s)	0.89±0.24	0.84±0.22	0.175
Velocity in the tricuspid regurgitation jet (m/s)	1.65±1.66	1.35±1.40	0.132

When patients who underwent surgery during the study are excluded, there was no statistically significant reduction in the flow rate through the mitral valve in the non-operated group (Screening value 1.23±0.28 vs Final study value 1.19±0.24; p=0.544)

Figure S1. Effects of orodispersible minitablets of enalapril on z score for systolic blood pressure (SBP)

A) By previous angiotensin-converting enzyme (ACE) inhibitor use

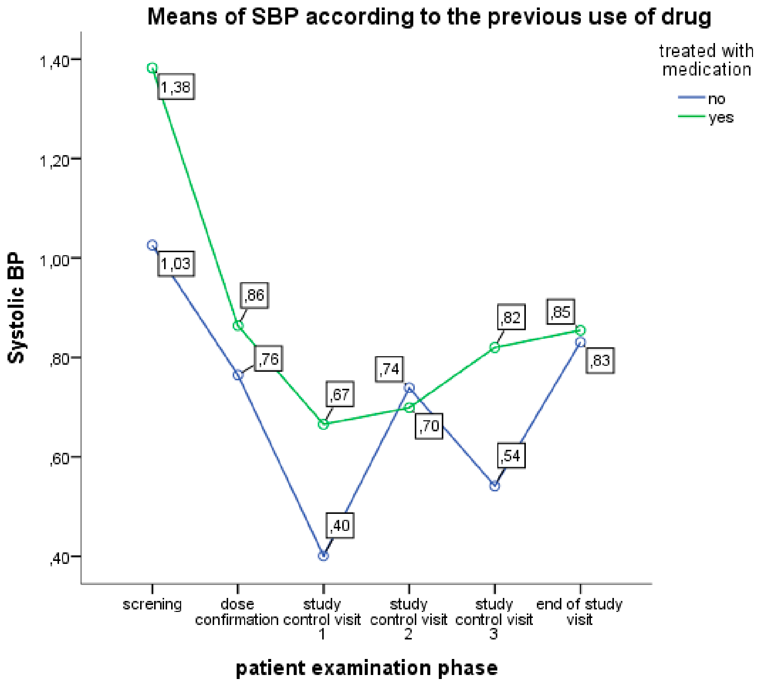

B) By age

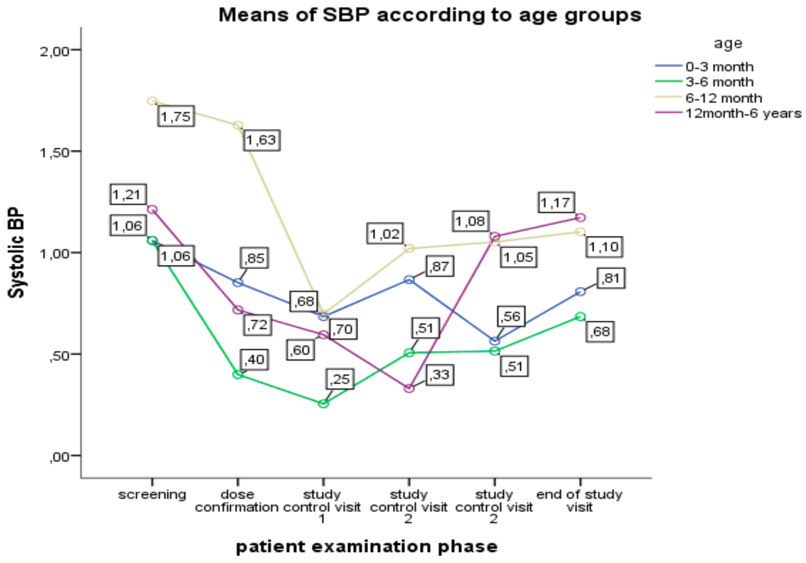

Figure S2. Effects of orodispersible minitablets of enalapril on z score for diastolic blood pressure (DBP)

A) By previous angiotensin-converting enzyme (ACE) inhibitor use

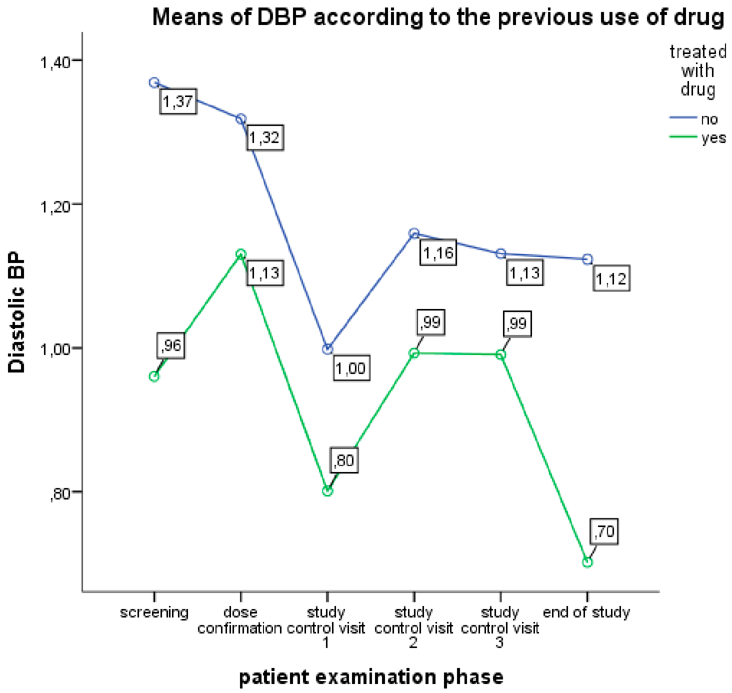

B) By age

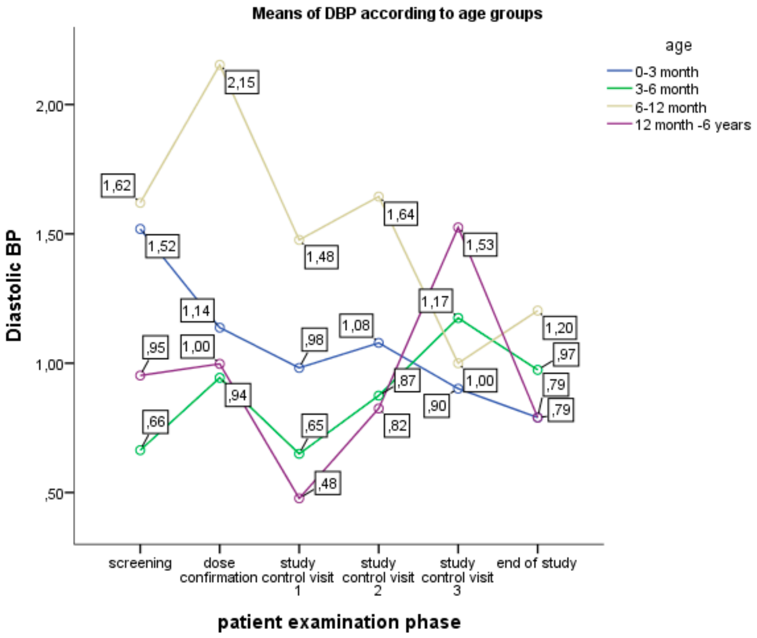

Figure S3. Effects of orodispersible minitablets of enalapril on heart rate (HR) in the initial phase in naïve and pretreated patients

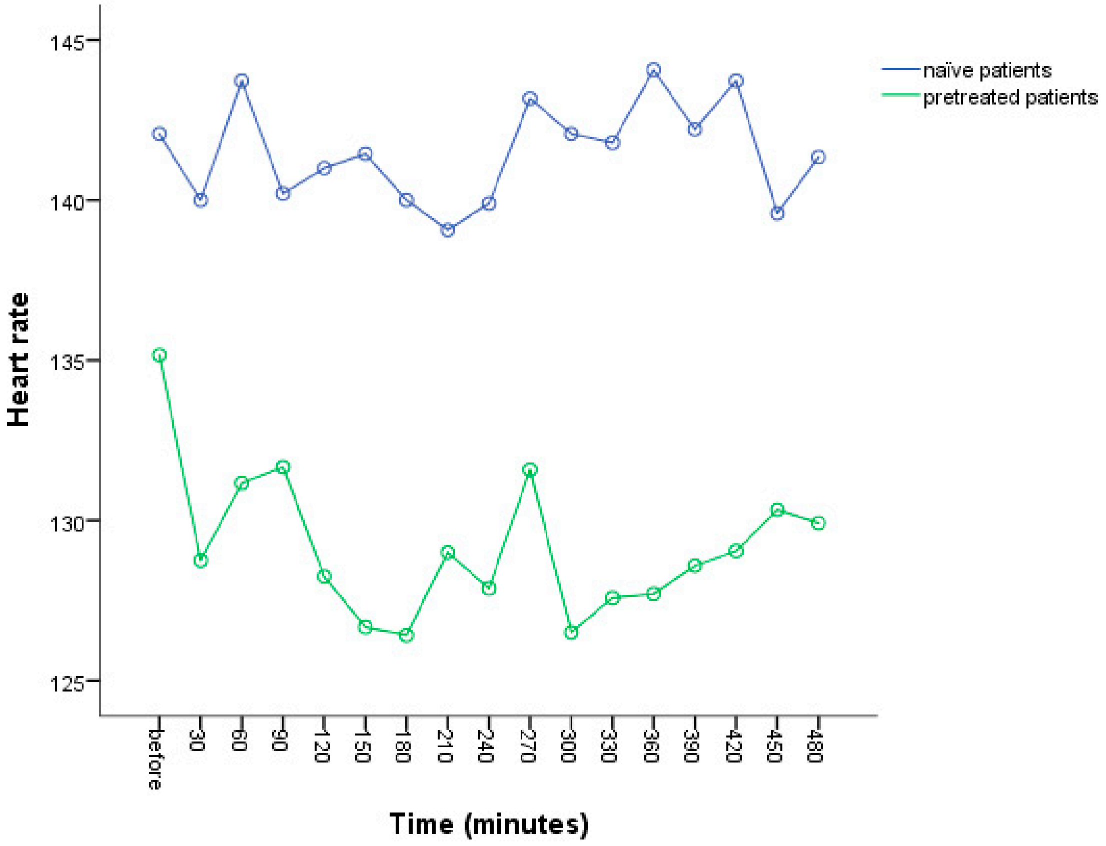

Figure S4. Effects of orodispersible minitablets of enalapril on heart rate (HR)

A) By previous angiotensin-converting enzyme (ACE) inhibitor use

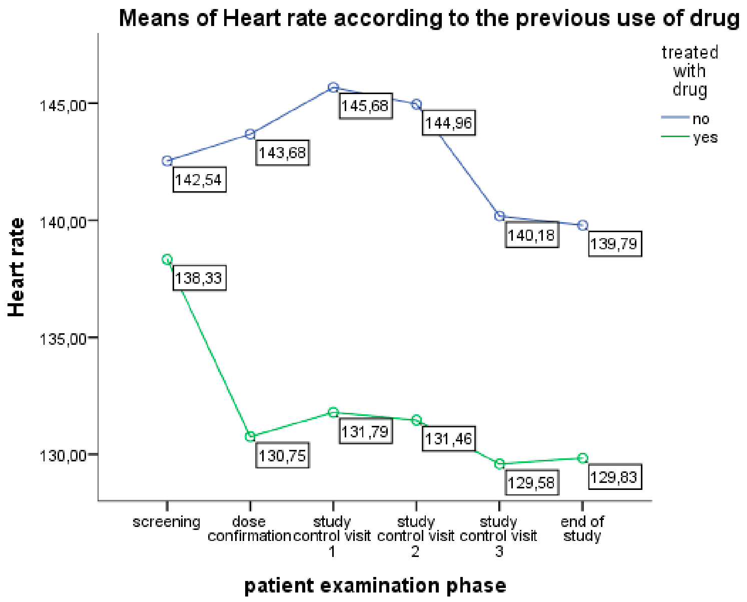

B) By age

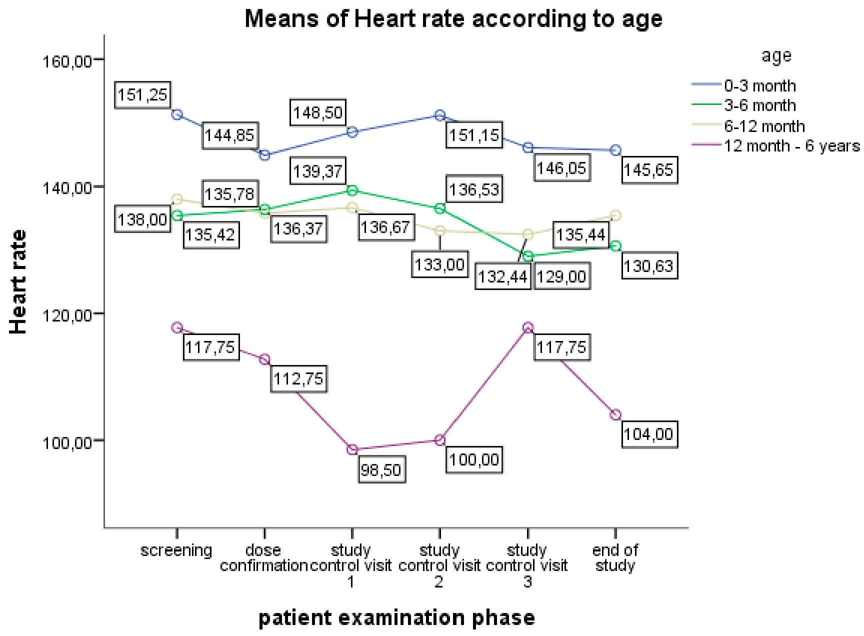

Supplement: Supplementary file 1 [file jcm-13-04976-s001.zip › jcm-3126569-supplementary.pdf]
